# Evidence of dissemination of a *clc*-type integrative and conjugative element to *Stenotrophomonas maltophilia*, mediating acquisition of *sul1* and other resistance determinants

**DOI:** 10.1128/aac.01554-24

**Published:** 2025-01-16

**Authors:** Selene Rebecca Boncompagni, Eleonora Riccobono, Maria Grazia Cusi, Vincenzo Di Pilato, Gian Maria Rossolini

**Affiliations:** 1 Department of Experimental and Clinical Medicine, University of Florence 415681 , Florence, Italy; 2 Microbiology and Virology Unit, Careggi University Hospital 18561 , Florence, Italy; 3 Department of Medical Biotechnologies, University of Siena 574285 , Siena, Italy; 4 Microbiology and Virology Unit, Siena University Hospital 161157 , Siena, Italy; 5 Department of Surgical Sciences and Integrated Diagnostics, University of Genoa 9302 , Genoa, Italy; 6 Microbiology Unit, IRCCS Ospedale Policlinico San Martino 9246 , Genoa, Italy; University of Fribourg, Fribourg, Switzerland

**Keywords:** ICE, mobile elements, VIM, antimicrobial resistance, *Pseudomonas*

## Abstract

A *Stenotrophomonas maltophilia* strain positive for the *bla*_VIM-1_ metallo-beta-lactamase gene and resistant to trimethoprim-sulfamethoxazole was unexpectedly isolated from a surveillance rectal swab. The characterization of the strain revealed carriage of a 91 kb integrative and conjugative element (ICE) harboring several resistance determinants [*sul1*, *bla*_VIM-1_, *aac(6')-Ib*, *aac(6')-31*, *qacE*∆*1*, *cld*, and *merEDAPTR*], closely related with a group of *clc*-type ICEs widespread among *Pseudomonas aeruginosa* and other pseudomonads. Results highlighted the possible spreading of similar elements to *S. maltophilia*, mediating the acquisition of relevant resistances.

## INTRODUCTION

*Stenotrophomonas maltophilia* is an opportunistic Gram-negative non-fermenting pathogen mostly causing infections in the healthcare setting and in subjects with chronic pulmonary conditions (e.g. cystic fibrosis and bronchiectasis) (1).

Treatment of *S. maltophilia* infections is challenging due to intrinsic resistance to several antimicrobials including aminoglycosides and β-lactams, mediated by several resistance mechanisms evolved in this species (e. g. aminoglycoside-modifying enzymes, efflux pumps, and resident β-lactamases) (2, 3). In particular, *S. maltophilia* produces two chromosomally encoded β-lactamases: the L1 metallo-β-lactamase (MBL) and the L2 extended-spectrum serine β-lactamase, which, taken together, can degrade most β-lactams (1, 3, 4).

Trimethoprim-sulphamethoxazole (SXT) remains among the first-line agent recommended for treating *S. maltophilia* infections (3, 5) but acquired resistance to this agent has been reported at variable rates (4%–21%) (6). SXT resistance is mainly due to the acquisition of *sul* genes encoding sulphonamide-resistant dihydropteroate synthetases (e.g. *sul1* and *sul2*) (7–10). Notably, *sul1* is typically found associated with class 1 integron platforms, but limited knowledge is available on the structure of the cognate genetic elements mediating *sul1* acquisition in *S. maltophilia*.

In this study, we report on the genotypic characterization of a *S. maltophilia* strain carrying a complex integrative and conjugative element (ICE), closely related to a group of ICEs that are widespread in *Pseudomonas aeruginosa* and other pseudomonads, which mediated acquisition of several resistance markers including *sul1*.

*S. maltophilia* AOUS-28640 was unexpectedly isolated, in September 2021, from a surveillance rectal swab taken from a patient admitted to the Siena University Hospital (central Italy) for a non-infectious condition. Information on previous antibiotic therapy was not available. During hospitalization, infections by *S. maltophilia* were not reported for the patient. Screening for carbapenemase genes in the surveillance rectal swab, taken at admission and performed using the Allplex Entero-DR assay (Seegene, Seoul, South Korea), yielded positivity for *bla*_VIM_, and subsequent culture of the sample on selective chromogenic medium (CHROMID CARBA-SMART, bioMérieux, Marcy-l'Étoile, France) yielded a Gram-negative rod identified as *S. maltophilia* by MALDI-ToF (MALDI Biotyper, Bruker Daltonics, Bremen, Germany). VIM-type carbapenemase production by *S. maltophilia* AOUS-28640 was confirmed with a Lateral Flow Immunoassay (RESIST-5 O.O.K.N.V., Coris Bioconcept, Gembloux, Belgium) (data not shown).

Antimicrobial susceptibility of *S. maltophilia* AOUS-28640 was tested by broth microdilution (BMD) using a commercial panel (ITGN E1-184-100, Merlin Diagnostika, Bornheim, Germany), or a custom panel (ThermoFisher, Waltham, MA, USA), or in-house reference BMD (11), as shown in Table 1. Chloramphenicol was from Sigma-Aldrich (St. Louis, MO, USA). Cefiderocol was from Shionogi (Osaka, Japan). For cefiderocol testing, an iron-depleted medium was used (12). Results were interpreted according to clinical breakpoints from EUCAST (v.14, [13]) or CLSI (M100-Ed34) (14).

**TABLE 1:** Antimicrobial susceptibility profile of *S. maltophilia* AOUS-28640^*a*^

Antimicrobial agent	MIC (mg/L)	Category^*b*^
Minocycline	0.5	(S)**
Levofloxacin	0.25	(S)**
Trimethoprim-sulfamethoxazole	>8/152	(R)*^/^**
Chloramphenicol	4	(S)**
Cefiderocol	0.25	(S)* ^/^**
Ceftazidime	16	N.A.
Ceftazidime/avibactam	16/4	N.A.
Meropenem	32	N.A.
Meropenem/vaborbactam	32/8	N.A.
Imipenem	>8	N.A.
Imipenem/relebactam	>8/4	N.A.
Aztreonam	>8	N.A.
Aztreonam/avibactam	1/4	N.A.
Amikacin	≤4	N.A.
Gentamicin	8	N.A.

^*a*^ Results were interpreted according to EUCAST (*) or CLSI (**) clinical breakpoints, when available.

^*b*^ N.A., not available.

Antimicrobial susceptibility testing revealed resistance to SXT and susceptibility to minocycline, levofloxacin, chloramphenicol, and cefiderocol. MIC of aztreonam/avibactam was 1/4 mg/L (i.e. lower than the EUCAST susceptibility breakpoint for *Enterobacterales*), while MIC of aztreonam alone was >8 mg/L. MICs of ceftazidime and meropenem were 16 and 32 mg/L, respectively, being unaffected by the presence of avibactam or vaborbactam (Table 1).

Whole-genome sequencing (WGS) analysis of *S. maltophilia* AOUS-28640 was performed using both Illumina MiSeq (Illumina Inc., San Diego, USA) and Nanopore MinION (Oxford Nanopore Technologies, Oxford, UK) platforms, as previously described (15). Comparative sequence analyses were performed by BLAST (https://blast.ncbi.nlm.nih.gov/Blast.cgi) and Mauve v.2.4.1. (https://github.com/koadman/mauve), using *S. maltophilia* NCTC10258 as a reference genome.

**Fig 1:**
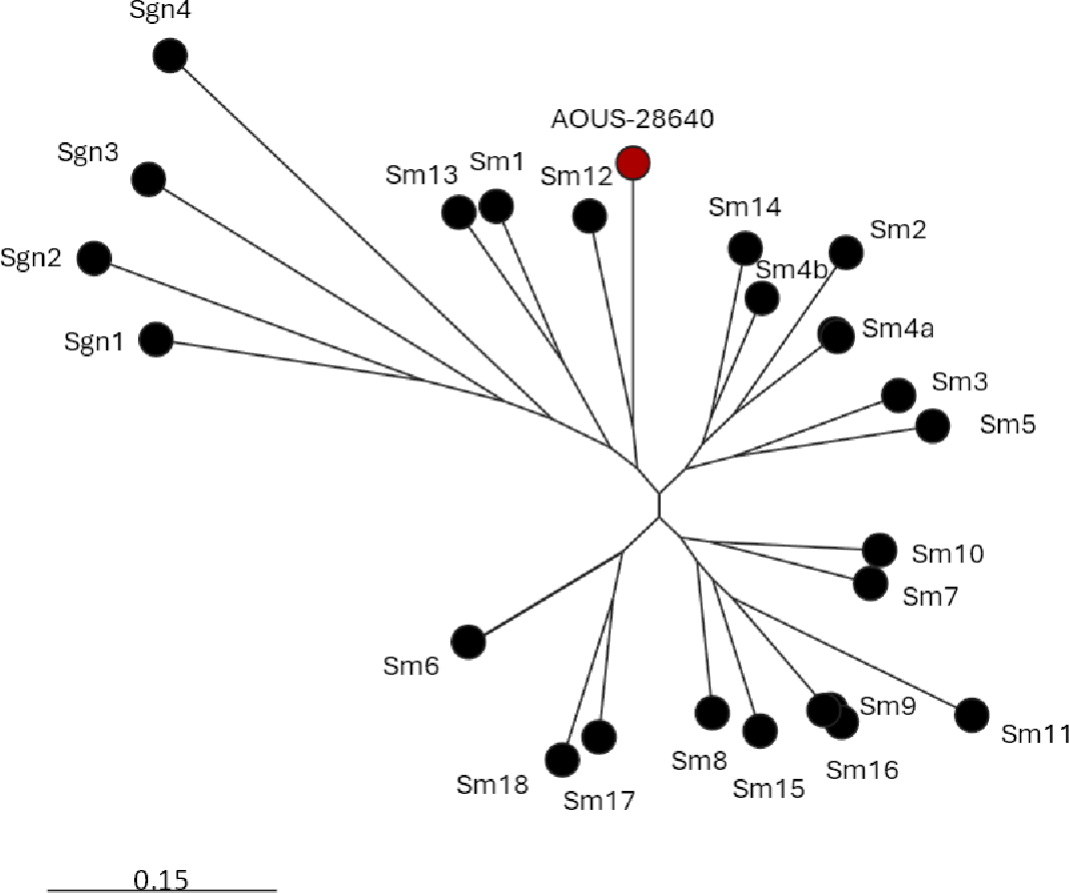
Phylogenomic analysis of *S. maltophilia* genomes, including representatives of the 23 monophyletic lineages of *S. maltophilia* complex (Table S2) previously described by Gröschel et al. (15), and *S. maltophilia* AOUS-28640 (red circle). Midpoint-rooted maximum-likelihood phylogenetic tree was built using a previously validated core-genome scheme (16).

WGS yielded a complete circular chromosome of 5,008,201 bp (GC%: 66.8) and confirmed species identification, while *in silico* MLST analysis (https://github.com/tseemann/mlst) revealed that AOUS-28640 belonged to a new sequence type, ST1228. Phylogenomic analysis, carried out as previously described using RAxML-NG with a GTR model and 1000 bootstrap replicates, and including previously recognized representatives of major *S. maltophilia* lineages as comparators (16), revealed that AOUS-28640 belonged to a new lineage, clustering with the Sm12 lineage which includes anthropogenic strains but is relatively distant from the Sm6 lineage (also known as *S. maltophilia sensu stricto*) (16) (Fig. 1).

Resistome profiling, carried out using AMRfinder (https://github.com/ncbi/amr), identified typical resident resistance determinants coding for aminoglycoside-modifying enzymes (*aph-3′-IIc* and *aac-6′-Iz*), multidrug efflux systems (*smeABC* and *smeDEF),* and the L1 and L2 β-lactamases (10). Notably, AOUS-28640 encoded an original variant of both L1, which was quite divergent from other variants, and L2 β-lactamase, showing a closer ancestry with the A, B, and C clades (4–17) (Fig. S1). Acquired resistance genes were also identified, including *aac(6')-Ib* and *aac(6')-31* encoding additional aminoglycoside-modifying enzymes, *bla*_VIM-1_, *sul1*, *qacE*∆1, *cld* (encoding a hypothetical chlorite dismutase), and a *merEDAPTR* module encoding a heavy metal detoxification system (Table 2).

**TABLE 2:** Content of acquired resistance genes in *S. maltophilia* strain AOUS-28640^*a*^

Gene	Identity	RefSeq nucleotide	Hosts species (strain)^*b*^
*bla*_VIM-1_	100%	NG_050336.1	*P. aeruginosa* (VR-143/97)
*aac(6')-Ib*	100%	NG_051844.1	*P. aeruginosa* (ED-1)
*aac(6')−31*	98.4%	NG_052214.1	*A. faecalis* (ZD02)
*sul1*	100%	NG_048099.1	*K. pneumoniae* (12836)
*qacE∆1*	100%	NG_048042.1	*P. aeruginosa* (N.A.)
*cld*	100%	MW595336.1	*P. aeruginosa* (NMI2634/13)
*merEDAPTR*	100%	CP128544.1	*P. mosselii* (NMI4849_14)

^*a*^ Identity (%) at the nucleotide level with most similar genes is also reported, together with source host species and strains information.

^*b*^ N.A., not available.

**Fig 2:**
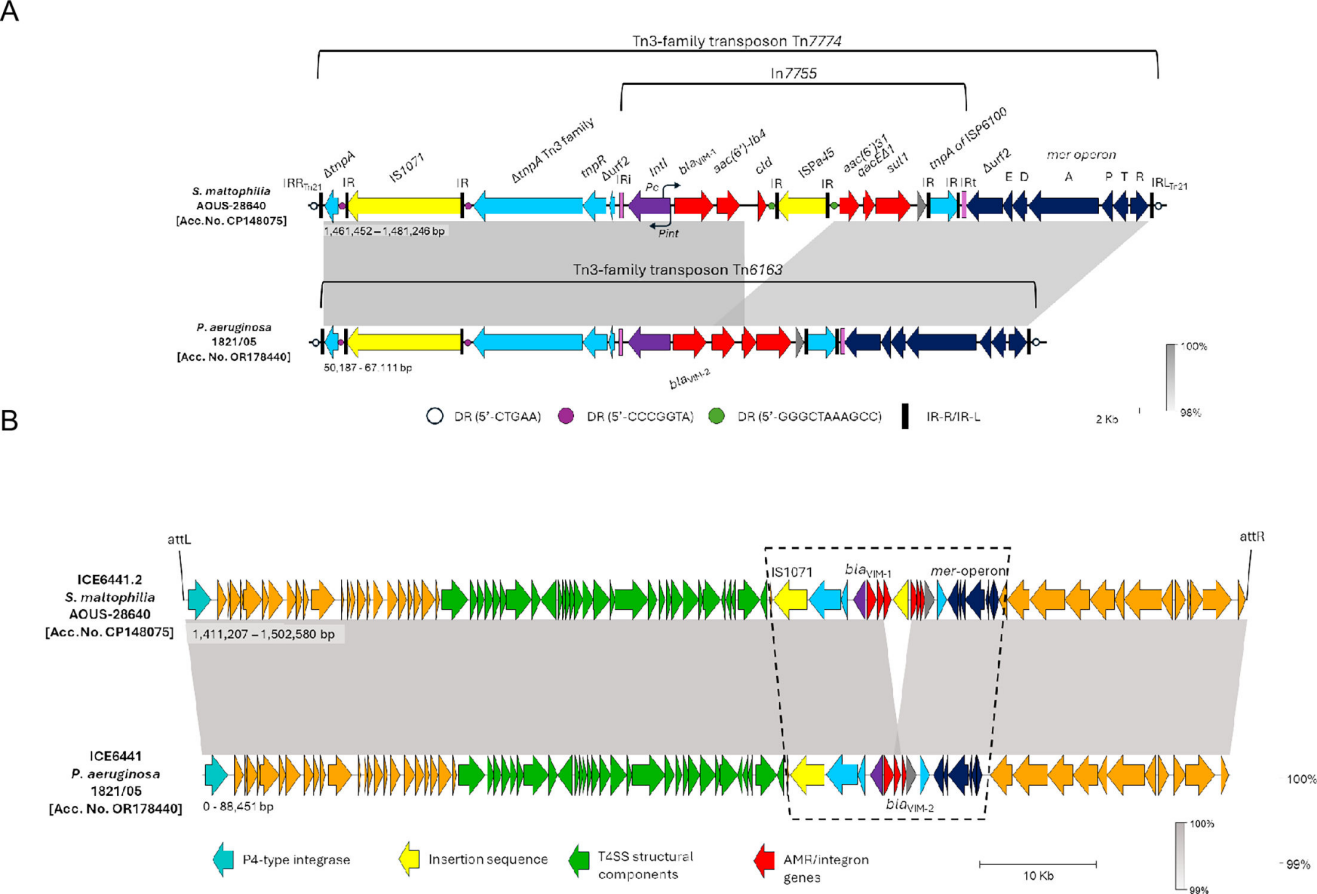
Schematic representation of the Tn*3*-family transposon Tn*7774*, carrying the acquired resistance genes of *S. maltophilia* AOUS-28640, and comparison with Tn*6163* (panel A). The structure of ICE*6441*.*2* from *S. maltophilia* AOUS-28640 and comparison with ICE*6441* from *P. aeruginosa* 1821/05 (panel B). Genes are shown as arrows of different colors according to the encoded function: drug resistances (red), insertion sequences (yellow), heavy metal detoxification system (dark blue), class one integron integrase (purple), ICE transfer modules (green), phage-related integrase (cyan), and others (orange). Direct repeats (DRs) are also shown in different colors according to the duplicated target sequence. The transposon elements (Tn*7774* and Tn*6163*) are boxed by a dashed line.

Analysis of the genetic context of the acquired resistance genes, carried out with the help of BLAST (https://blast.ncbi.nlm.nih.gov/Blast.cgi), ICEFinder (https://bioinfo-mml.sjtu.edu.cn/ICEfinder/), Integronfinder (https://github.com/gem-pasteur/Integron_Finder), and ISFinder (https://isfinder.biotoul.fr/), revealed that they were all embedded in a Tn*3*-like transposon (Tn*7774*) which carried the *merEDAPTR* module and a class 1 integron (In*7755*) to which the other resistance genes were associated. Transposon Tn*7774* was closely related to Tn*6163* from *P. aeruginosa* 1821/05, a multidrug-resistant clinical strain from Poland producing the VIM-2 MBL (18) (Fig. 2A). The analysis of the regions flanking Tn*7774* revealed that it was inserted into a large ICE of the *clc*-family (19), very similar (99.9% nucleotide identity, 96% of query coverage) to ICE*6441,* the *clc*-like ICE carrying Tn*6163* (Fig. 2B) (18), and was, hence, named ICE*6441*.2. Interestingly, ICE*6441*.2 was inserted at the 3′-end of a chromosomal tRNA^Gly^ gene (corresponding to locus tag DQN92_RS16355 in the *S. maltophilia* NCTC10258 reference genome), suggesting that this site could function as a target for ICE integration in the *S. maltophilia* chromosome, as it does in *P. aeruginosa* (18).

A search of the NCBI database (carried out on 1 December 2024, including only complete bacterial genomes) revealed the presence of ICE*6441*-like elements (≥99.2% nucleotide identity and ≥91% query coverage) in several strains of *P. aeruginosa* and other *Pseudomonas* species (including *P. monteilii*, *P. alloputida*, *P. asiatica*, *P. putida*, and *P. mosselii*) from various continents (Europe, Asia, and Australia) in one strain of *Aeromonas caviae*, in one strain of *Alcaligenes faecalis*, and in six strains of *S. maltophilia* (Table S1). Some of these latter were from large-scale sequencing studies involving environmental isolates from hospital settings, which did not focus on the presence of ICEs in *Stenotrophomonas* (20). Altogether, our findings revealed that large *clc*-type ICEs widespread among *Pseudomonas* can also spread to *S. maltophilia*, mediating the acquisition of relevant resistance determinants, including *sul1*. Transfer of a putative *clc*-type ICE (ICE*nah*CSV86) carrying genes for the utilization of aromatic hydrocarbons from *Pseudomonas bharatica* to *S. maltophilia* has previously been hypothesized (21, 22), although confirmatory WGS data for the *S. maltophilia* transconjugants are not available.

Genomic surveillance of *S. maltophilia* circulating in the clinical setting will be of interest to monitor this phenomenon, while mobilization of these elements to and from *S. maltophilia* deserves further investigation.

## Data Availability

The complete genome of *S. maltophilia* AOUS-28640 was deposited in GenBank (accession number CP148075). Novel elements were deposited and numbered according to the Transposon Registry (https://transposon.lstmed.ac.uk/).

## SUPPLEMENTAL MATERIAL

Supplemental material aac.01554-24-s0001.docx Figure S1; Tables S1 and S2.

ASM does not own the copyrights to Supplemental Material that may be linked to, or accessed through, an article. The authors have granted ASM a non-exclusive, world-wide license to publish the Supplemental Material files. Please contact the corresponding author directly for reuse.
