# Supplementary material for: Evidence of dissemination of a clc-type integrative and conjugative element to Stenotrophomonas maltophilia, mediating acquisition of sul1 and other resistance determinants
Source: Antimicrob Agents Chemother. 2025 Jan 16;69(2):e01554-24. doi: 10.1128/aac.01554-24 (PMC11823659; doi:10.1128/aac.01554-24)
Supplement: Supplemental material — Figure S1; Tables S1 and S2. [file aac.01554-24-s0001.docx]

**Figure S1. Comparative analysis of L1 (n=88, panel A) and of L2 (n=119, panel B) β-lactamase sequences of *S. maltophilia* and other *Stenotrophomonas* species. Unrooted circular cladograms incudes sequences previously analyzed by Mojica *et al*. (1) plus L1 and L2 sequences of *S. maltophilia* AOUS-28640, and L1-like enzymes described in other *Stenotrophomonas* species (*S. lactitubi*, *S. pavanii*, *S. indicatrix*, *S. geniculata*) (2). Alignment was generated using Clustal Omega, and cladogram visualized in Microreact (https://microreact.org/). Branch length is proportional to the protein sequence divergence, with the scale bar indicating the number of substitutions per residue. Clades of L1 β-lactamase (n=5) are highlighted by capital letters (A-E) and with different colours. Clades of L2 β-lactamase (n=4) are highlighted by capital letters (A-D) and with different colours.**

E

AOUS-28640

*S. indicatrix*

*S. lactitubi*

A

*S. geniculata*

*S. pavanii*

D

B

C


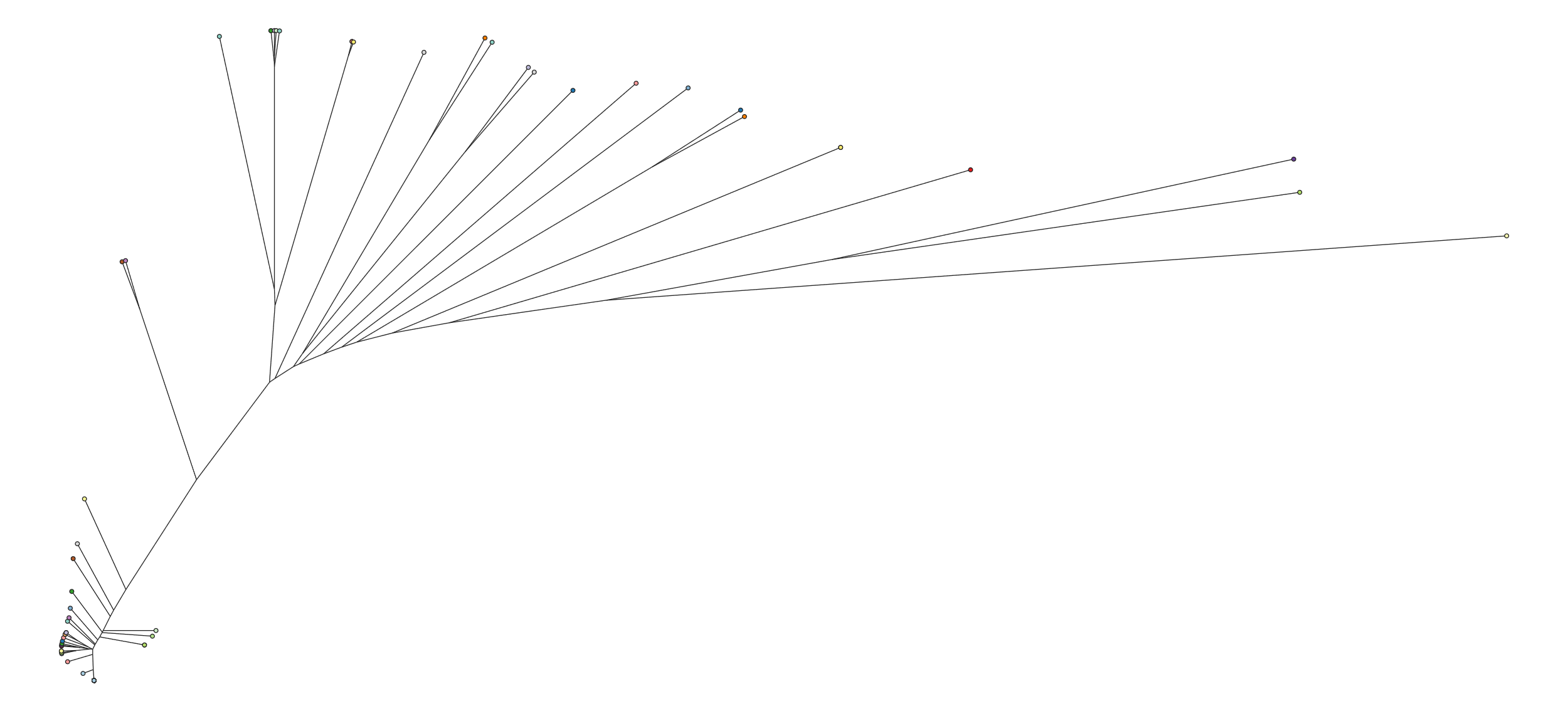


0.015

A

AOUS-28640

D

A

C

B


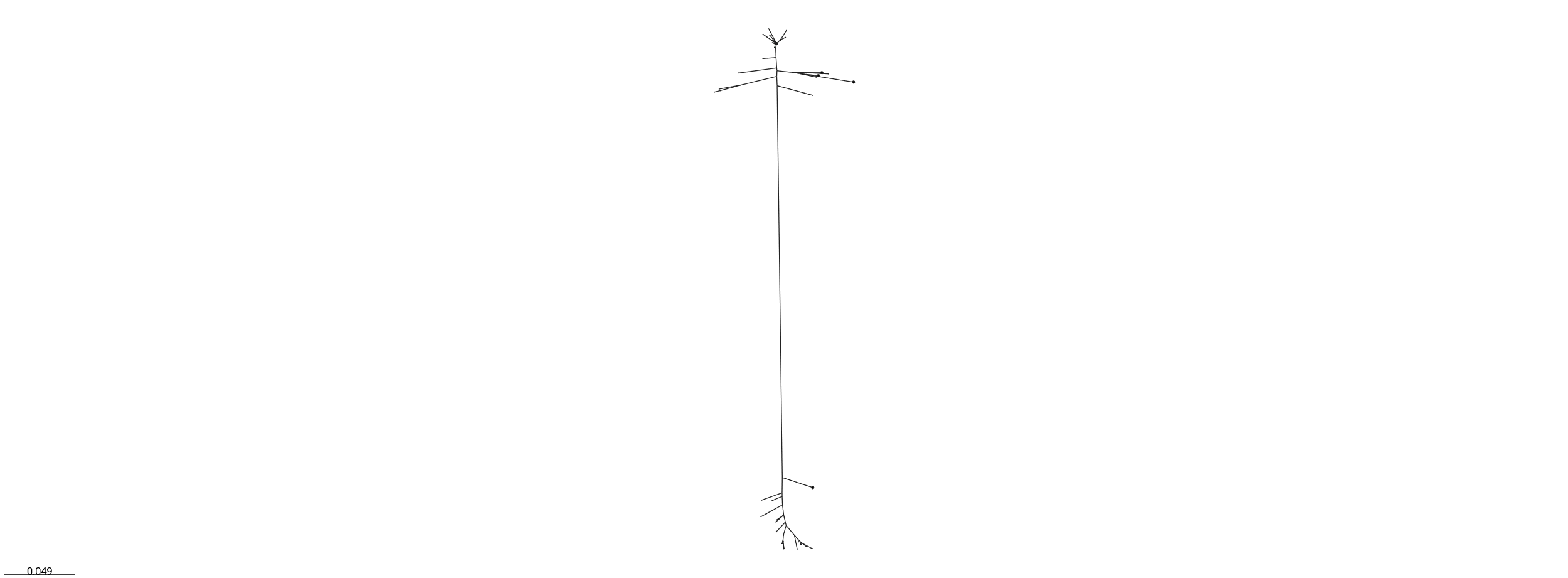

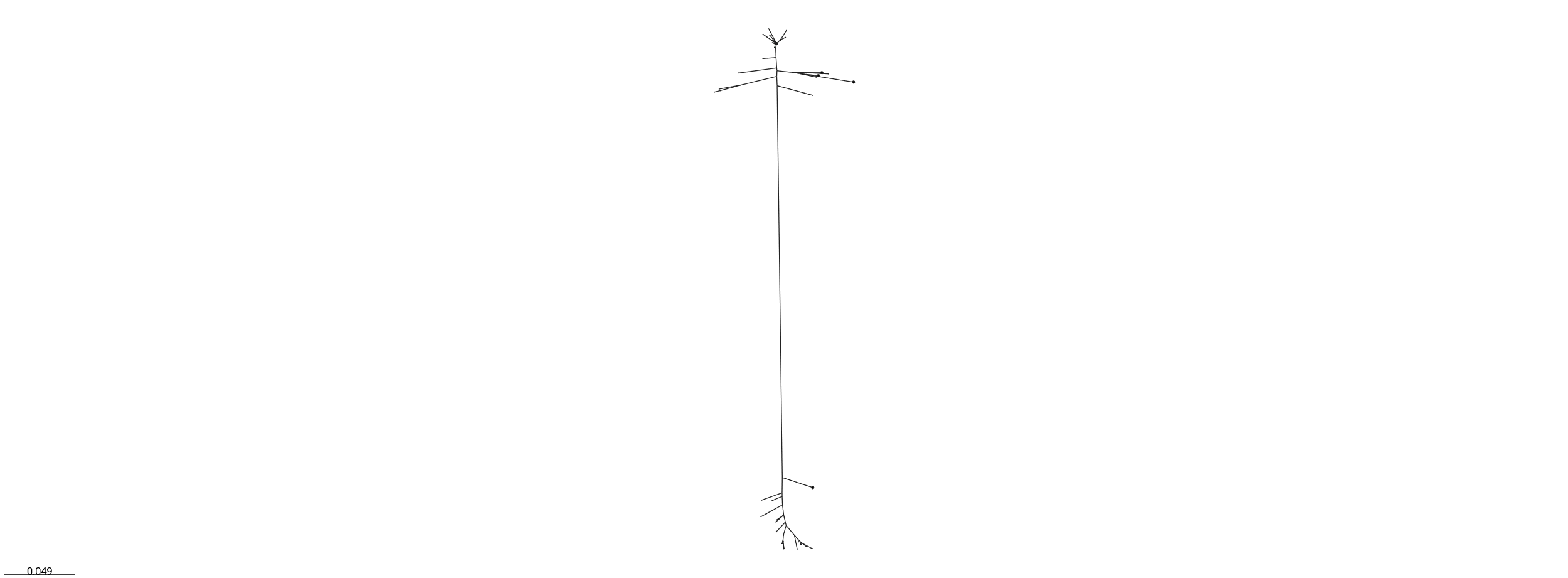


B

**Table S1. Summary of all records identified in the NCBI database with ≥99.2% nucleotide identity and ≥91% query coverage to ICE*6441*-like elements (carried out on December 01, 2024 including only complete genomes).**

| **Strain** | **Species** | **Query Coverage (%)** | **Identity (%)** | **Geographic location#** | **Accession** |
| --- | --- | --- | --- | --- | --- |
| NMI4849_14 | Pseudomonas mosselii | 96% | 99.9 | Poland | CP128544.1 |
| FIDG-26323 | Pseudomonas aeruginosa | 96% | 99.9 | Italy | CP150883.1 |
| GD04089 | Stenotrophomonas malthopilia | 96% | 99.4 | Pakistan | JAOEBI010000006.1^3^ |
| GD03794 | Stenotrophomonas malthopilia | 96% | 99.4 | Pakistan | JAOCFV010000007.1^3^ |
| PL12 | Stenotrophomonas malthopilia | 96% | 99.4 | Slovenia | RAUP01000012.1^4^ |
| OL11 | Stenotrophomonas malthopilia | 96% | 99.4 | Slovenia | RAUO01000013.1^4^ |
| A1_134092 | Stenotrophomonas malthopilia | 94% | 99.5 | France | CABFZQ020000026.1 |
| ARGA00101 | Stenotrophomonas malthopilia | 94% | 99.5 | Spain | DAZYXA010000007.1 |
| CCUG 51971 | Pseudomonas aeruginosa | 93% | 99.9 | Sweden | CP043328.1 |
| NMI3658_15 | Pseudomonas asiatica | 93% | 99.9 | Poland | CP128546.1 |
| RIVM-EMC2982 | Pseudomonas aeruginosa | 93% | 99.9 | Netherlands | CP016955.1 |
| NY5051 | Pseudomonas aeruginosa | 93% | 99.9 | China | CP120707.1 |
| Carb01 63 | Pseudomonas aeruginosa | 93% | 99.9 | Netherlands | CP011317.1 |
| NMI2351/13 | Pseudomonas aeruginosa | 93% | 99.8 | Poland | CP128277.1 |
| 6762 | Pseudomonas aeruginosa | 93% | 99.8 | China | CP030075.1 |
| NMI2658/07 | Pseudomonas aeruginosa | 93% | 99.4 | Poland | CP128279.1 |
| 8712/11 | Pseudomonas monteilii | 92% | 99.9 | Poland | OR178455.1 |
| PaLo538 | Pseudomonas aeruginosa | 92% | 99.9 | Germany | CP075766.1 |
| F291007 | Pseudomonas aeruginosa | 92% | 99.9 | China | CP081345.1 |
| SE5352 | Pseudomonas aeruginosa | 92% | 99.9 | China | CP054843.1 |
| 1,9E+09 | Pseudomonas aeruginosa | 92% | 99.9 | China | CP060392.1 |
| NY11254 | Pseudomonas aeruginosa | 92% | 99.9 | China | CP096960.1 |
| NY5523 | Pseudomonas aeruginosa | 92% | 99.9 | China | CP096941.1 |
| PA5083 | Pseudomonas aeruginosa | 92% | 99.9 | China | CP102174.1 |
| 3658/15 | Pseudomonas asiatica | 92% | 99.9 | Poland | OR178459.1 |
| 6266/12 | Pseudomonas monteilii | 92% | 99.9 | Poland | OR178457.1 |
| FI-14/157 | Pseudomonas aeruginosa | 92% | 99.9 | Italy | CP133737.1 |
| 2426/06 | Pseudomonas aeruginosa | 92% | 99.9 | Poland | OR178446.1 |
| Paer3285 | Pseudomonas aeruginosa | 92% | 99.9 | Bulgaria | CP135097.1 |
| CPO506 | Pseudomonas aeruginosa | 92% | 99.9 | Australia | CP143649.1 |
| CPO100 | Pseudomonas aeruginosa | 92% | 99.9 | Australia | CP143645.1 |
| CPO459 | Pseudomonas aeruginosa | 92% | 99.9 | Australia | CP143648.1 |
| CPO180 | Pseudomonas aeruginosa | 92% | 99.9 | Australia | CP143647.1 |
| CPO174 | Pseudomonas aeruginosa | 92% | 99.9 | Australia | CP143646.1 |
| PSA9 | Pseudomonas aeruginosa | 92% | 99.9 | Italy | CP150132.1 |
| SE5331 | Pseudomonas aeruginosa | 92% | 99.9 | China | CP046402.2 |
| FI-17645 | Pseudomonas aeruginosa | 92% | 99.9 | Italy | CP133738.1 |
| HPA1406 | Pseudomonas aeruginosa | 92% | 99.9 | South Korea | CP137491.1 |
| HPA0118 | Pseudomonas aeruginosa | 92% | 99.9 | South Korea | CP137522.1 |
| HPA0384 | Pseudomonas aeruginosa | 92% | 99.9 | South Korea | CP137500.1 |
| NMI8712_11 | Pseudomonas monteilii | 92% | 99.9 | Poland | CP128546.1 |
| HPA0044 | Pseudomonas aeruginosa | 92% | 99.9 | South Korea | CP137505.1 |
| NMI6266_12 | Pseudomonas monteilii | 92% | 99.9 | Poland | CP128545.1 |
| PA1020 | Pseudomonas aeruginosa | 92% | 99.9 | China | CP133397.1 |
| 6180/09 | Pseudomonas asiatica | 92% | 99.9 | Poland | OR178458.1 |
| 2351/13 | Pseudomonas aeruginosa | 92% | 99.8 | Poland | OR178443.1 |
| MUB14 | Alcaligenes faecalis | 92% | 99.4 | Poland | CP048039.1 |
| 2658/07 | Pseudomonas aeruginosa | 92% | 99.4 | Poland | OR178444.1 |
| AR442 | Pseudomonas aeruginosa | 91% | 99.9 | N.A. | CP029090.1 |
| 30094cz | Pseudomonas aeruginosa | 91% | 99.9 | Czech Republic | KY860573.1 |
| KAM376 | Aeromonas caviae | 91% | 99.9 | Japan | AP024402.1 |
| LRJ32 | Pseudomonas aeruginosa | 91% | 99.9 | Denmark | CP115191.1 |
| DHS01 | Pseudomonas aeruginosa | 91% | 99.9 | France | CP013993.1 |
| pae001 | Pseudomonas aeruginosa | 91% | 99.9 | China | CP133094.1 |
| NY5532 | Pseudomonas aeruginosa | 91% | 99.9 | China | CP096950.1 |
| MRSN365855 | Pseudomonas putida | 91% | 99.9 | Thailand | CP132007.1 |
| Y82 | Pseudomonas aeruginosa | 91% | 99.9 | South Korea | CP030912.1 |
| 3088/05 ICE6441-PL9 | Pseudomonas alloputida | 91% | 99.8 | Poland | OR178448.1 |
| 2291/10 | Pseudomonas alloputida | 91% | 99.8 | Poland | OR178450.1 |
| 2153/05 ICE6441-PL8 | Pseudomonas alloputida | 91% | 99.8 | Poland | OR178447.1 |
|  |  |  |  |  |  |

**^#^: N.A., not available**

**Table S2. Accession number of a total of 23 representatives’ genomes of *S. maltophilia* used for phylogenomic analysis (5).**

| **Lineages** | **Accession number** |
| --- | --- |
|  |  |
| Sgn1 | GCF_002025605.1 |
| Sgn2 | GCA_002377295.1 |
| Sgn3 | ERR1974548 |
| Sgn4 | GCF_001676315.1 |
| Sm1 | GCF_001431665.1 |
| Sm2 | ERR3300139 |
| Sm3 | ERR1974500 |
| Sm4a | GCF_000284595.1 |
| Sm4b | GCF_002847385.1 |
| Sm5 | ERR33000248 |
| Sm6 | GCF_000072485.1 |
| Sm7 | ERR3299886 |
| Sm8 | GCF_002138415.1 |
| Sm9 | ERR3300012 |
| Sm10 | GCF_001274655.1 |
| Sm11 | ERR3299834 |
| Sm12 | GCF_001274595.1 |
| Sm13 | ERR33006245 |
| Sm14 | GCF_00279245.1 |
| Sm15 | ERR3300146 |
| Sm16 | ERR3299978 |
| Sm17 | ERR3300026 |
| Sm18 | GCF_002798925.1 |

**References**

1. Mojica MF, Rutter JD, Taracila MAbriata LAFouts DE, Papp-Wallace KMWalsh TJ, LiPuma JJ, Vila AJ, Bonomo RA. 2019. Population Structure, Molecular Epidemiology, and β-Lactamase Diversity among *Stenotrophomonas maltophilia* Isolates in the United States. mBio e00405-19. https://doi.org/10.1128/mBio.00405-19.
2. Yamada K, Ishii Y, Tateda K. 2024. Biochemical characterization of the L1-like metallo-β-lactamase from *Stenotrophomonas lactitubi*. Antimicrobial agents and chemotherapy, 68(3), e0086623. <https://doi.org/10.1128/aac.00866-23>.
3. Mustapha, M. M., Srinivasa, V. R., Griffith, M. P., Cho, S. T., Evans, D. R., Waggle, K., Ezeonwuka, C., Snyder, D. J., Marsh, J. W., Harrison, L. H., Cooper, V. S., & Van Tyne, D. 2022. Genomic Diversity of Hospital-Acquired Infections Revealed through Prospective Whole-Genome Sequencing-Based Surveillance. mSystems, 7(3), e0138421.
4. Yero D, Huedo P, Conchillo-Solé O, et al. Genetic Variants of the DSF Quorum Sensing System in *Stenotrophomonas maltophilia* Influence Virulence and Resistance Phenotypes Among Genotypically Diverse Clinical Isolates. *Front Microbiol*. 2020;11:1160.
5. Gröschel MI, Meehan CJ, Barilar I, Diricks M, Gonzaga A, Steglich M, Conchillo-Solé O, Scherer IC, Mamat U, Luz CF, De Bruyne K, Utpatel C, Yero D, Gibert I, Daura X, Kampmeier S, Rahman NA, Kresken M, van der Werf TS, Alio I, Kohl TA. 2020. The phylogenetic landscape and nosocomial spread of the multidrug-resistant opportunist *Stenotrophomonas maltophilia*. Nat Commun 11(1), 2044. https://doi.org/10.1038/s41467-020-15123-0.
